# Syrian Medical Association

**Published:** 1842-04

**Authors:** 


### Syrian Medical-Aid Association.

A SOCIETY has recently been established under this name, chiefly through the benevolent exertions of Sir Culling Smith and Dr. Hodgkin. Its nature and objects, and its high claims on the sympathy and co-operation of the rich and charitable, will be sufficiently manifest from the following brief extract from a prospectus issued by the Association:

"Of all the blessings which England might, out of her abundance, impart to the Syrians—the one which they most desire and appreciate—is medical skill. The natives who profess medicine are utterly ignorant of the science, and are, generally, of the meanest capacity. The poor people all over the country are in sad misery, for want of medical advice; and they flock to such travellers as are in company of a physician. There is no such thing as a medical man in all Syria; nor do the people appear to have any local knowledge of herbs even. No hospitals, dispensaries, infirmaries, or public charities exist, to receive the diseased, the mendicant, the orphan, or the helpless widow. Sympathy can but offer prayer and hope; while sickness preys unchecked upon its victims, without science to eradicate, or benevolence to alleviate it.

"To alleviate these painful circumstances, a society has recently been formed, entitled the 'Syrian Medical-Aid Association,' for the purpose of sending out in the first instance, a surgeon or physician, with an assistant, and a store of drugs, &c. It is proposed that he shall visit the indigent sick, gratuitously. While it is not intended that he should make any direct missionary effort, it is considered indispensable that he should be a person of a decidedly Christian character; and who would thankfully avail himself of every opportunity to invite those, whom he might find favorably disposed, to have recourse to the Saviour—the physician of souls. It is hoped that he will thus become a valuable pioneer to the labours of Assaad Kayat, and other agents of the Syrian-Education or kindred Societies; the local knowledge of whom will open to him continual opportunities of usefulness.

"In this manner it is anticipated, that, under the providence of God—

"I. Much suffering and misery will be relieved; and one of the greatest of earthly blessings will be conferred upon the Syrian people.

"II. Our national character will be favorably exhibited. In the place where England's voice was lately heard in the roar of cannon and the cries of the wounded, it will now find its way to the bedside of the sick and dying, in the gentle accents of pity and relief.

"III. This manifestation of Christian benevolence may win a favorable hearing for those who shall come to teach the divine principles, of which such a practical exemplification will have been thus presented.

"IV. A benefit will be conferred on resident European merchants, and travellers; many of whom fall victims to disease, for want of competent medical advice. The medical agent will be allowed to receive payment for services rendered to these classes.

"To these benefits may be added the probable advantages of more closely studying, with the view to prevention and treatment, the plague, and other terrible diseases, which yearly destroy thousands in Syria and the adjoining countries."

